# Unsupervised machine learning combined with 4D scanning transmission electron microscopy for bimodal nanostructural analysis

**DOI:** 10.1038/s41598-024-53289-5

**Published:** 2024-02-05

**Authors:** Koji Kimoto, Jun Kikkawa, Koji Harano, Ovidiu Cretu, Yuki Shibazaki, Fumihiko Uesugi

**Affiliations:** 1 Center for Basic Research On Materials, National Institute for Materials Science (NIMS), 1-1 Namiki, Tsukuba, Ibaraki 305-0044 Japan; 2 Institute of Materials Structure Science, High Energy Accelerator Research Organization, Tsukuba, Japan; 3 Research Network and Facility Service Division, National Institute for Materials Science, Tsukuba, Japan

**Keywords:** Transmission electron microscopy, Characterization and analytical techniques, Nanoscale materials, Imaging techniques, Transmission electron microscopy, Metals and alloys

## Abstract

Unsupervised machine learning techniques have been combined with scanning transmission electron microscopy (STEM) to enable comprehensive crystal structure analysis with nanometer spatial resolution. In this study, we investigated large-scale data obtained by four-dimensional (4D) STEM using dimensionality reduction techniques such as non-negative matrix factorization (NMF) and hierarchical clustering with various optimization methods. We developed software scripts incorporating knowledge of electron diffraction and STEM imaging for data preprocessing, NMF, and hierarchical clustering. Hierarchical clustering was performed using cross-correlation instead of conventional Euclidean distances, resulting in rotation-corrected diffractions and shift-corrected maps of major components. An experimental analysis was conducted on a high-pressure-annealed metallic glass, Zr-Cu-Al, revealing an amorphous matrix and crystalline precipitates with an average diameter of approximately 7 nm, which were challenging to detect using conventional STEM techniques. Combining 4D-STEM and optimized unsupervised machine learning enables comprehensive bimodal (i.e., spatial and reciprocal) analyses of material nanostructures.

## Introduction

**Figure 1:**
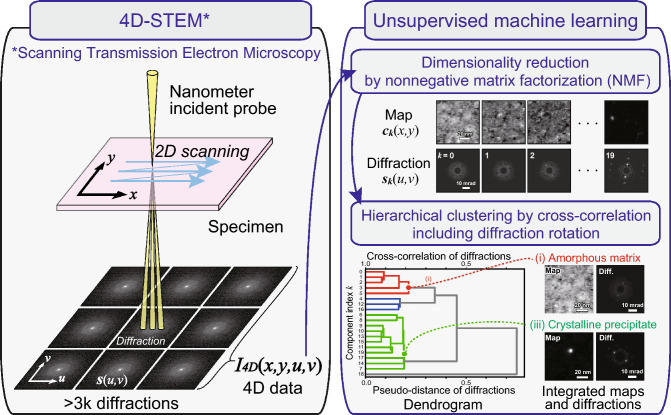
Schematic illustrating the combination of 4D-STEM and unsupervised machine learning in this study.

Modern scientific instrumentation techniques, such as scanning transmission electron microscopy (STEM), for material characterization can provide significantly larger experimental datasets than ever before. Four-dimensional (4D)-STEM^1–7^ has recently been achieved by recording spatially-resolved electron diffractions $s(u,v)$ with varying positions (*x*, *y*) of an incident probe, yielding 4D data ${I}_{4D}(x, y, u, v)$, as illustrated in Fig. 1. Electron diffraction with a nanometer incident probe can be used to crystallographically characterize inhomogeneous materials^8^. 4D-STEM provides bimodal information from real (*x*, *y*) and reciprocal (*u*, *v*) spaces as microscopic maps and diffraction patterns, respectively. Because the data acquired by 4D-STEM can easily reach 1 GB (e.g., 128^4^ pixels with 4 bytes per pixel), statistical or computational approaches are indispensable to extract representative information. Electron microscopists have applied statistical^3,5^ or machine learning techniques^2,4,9^ to large experimental datasets. Such techniques include principal component analysis (PCA)^10,11^ and non-negative matrix factorization (NMF)^12–14^ for denoising, dimensionality reduction, and other tasks. Various software packages are available, including well-known machine learning libraries (e.g., scikit-learn) or packages dedicated to electron microscopy (e.g., py4DSTEM)^15^. Although several machine learning techniques can be applied using general software packages, in-depth discussion or optimization based on knowledge of electron microscopy has hardly been addressed.

Various applications of machine learning to 4D-STEM have been reported^16^. Similarly, we have reported on strain mapping^17^, atomic resolution^18^, and combining 4D-STEM with NMF to extract interpretable diffraction patterns^19^ from composite specimens of known crystalline materials. Since the investigated specimens often have unknown structures, we need to elucidate the experimental results without learning supervision.

In this study, we combined 4D-STEM with unsupervised machine learning, including dimensionality reduction and hierarchical clustering (Fig. 1). We prepared in-house scripts (i.e., macros) in software for electron microscopy (DigitalMicrograph, Gatan^20^) for data preprocessing, NMF, and hierarchical clustering while implementing electron microscopy knowledge in its data processing and analyses. This study aimed to combine optimized machine learning with material characterization to achieve bimodal (i.e., image and diffraction) crystallographic analysis at the nanometer scale.

## Results

### Outline of dimensionality reduction by NMF

An experimental diffraction pattern of actual materials is often a linear combination of multiple diffractions from overlapping domains; therefore, factorization and dimensionality reduction should be applied before clustering. We applied NMF for dimensionality reduction to experimental 4D-STEM data, which consisted of 3364 diffractions (see METHODS). Among the available dimensionality reduction techniques, we found that NMF provides interpretable results (electron diffractions), whereas the well-known PCA is ineffective for dimensionality reduction or component number estimation for 4D-STEM data^19^.

**Figure 2:**
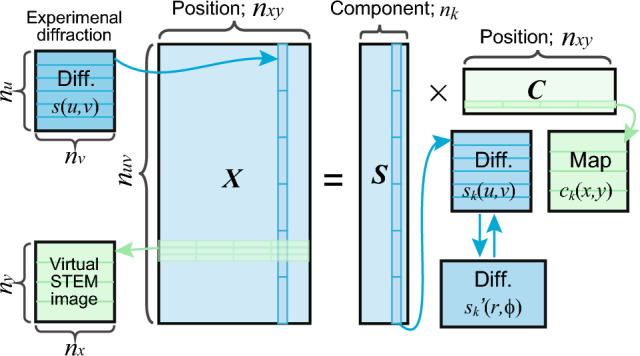
Schematic illustrating the dimensionality reduction using NMF for 4D-STEM.

Dimensionality reduction by NMF represents the experimental data ***X*** as a linear combination of diffractions ***S***, consisting of sparse positive diffractions as follows (Fig. 2):

1 $${\varvec{X}}={\varvec{S}} {\varvec{C}},$$

where ***C*** represents positive coefficients in real space, called maps in this article. If the maximum number of each coordinate tuple in the experiment is $\left({n}_{x},{n}_{y},{n}_{u},{n}_{v}\right)$ and the total number of components is *n*_*k*_ (*n*_k_ <  < *n*_*xy*_), then ${\varvec{X}}\in {\mathbb{R}}_{+}^{{n}_{uv}\times {n}_{xy}}$, ${\varvec{S}}\in {\mathbb{R}}_{+}^{{n}_{uv}\times {n}_{k}}$, and ${\varvec{C}}\in {\mathbb{R}}_{+}^{{n}_{k}\times {n}_{xy}}$, where ${n}_{xy}={n}_{x}\times {n}_{y}$ and ${n}_{uv}={n}_{u}\times {n}_{v}$. This transformation from 4D data ${{\varvec{I}}}_{4{\varvec{D}}}\left(x,y,u,v\right)$ to two-dimensional (2D) matrix ${\varvec{X}}$ is necessary for matrix calculations in NMF, and the experimental diffraction pattern ${\varvec{s}}\left(u,v\right)$ at each position is transformed into one-dimensional (1D) column vectors of ***X***. NMF yields real-space maps ${{\varvec{c}}}_{{\varvec{k}}}\left(x,y\right)$ and reciprocal-space diffractions ${{\varvec{s}}}_{{\varvec{k}}}\left(u,v\right)$ as row and column vectors of ***C*** and ***S***, respectively (Fig. 2). Owing to this data transformation, various material information (e.g., domain sizes in real space and lattice parameters in reciprocal space) is not handled, and the bimodal information in 4D-STEM cannot be used during NMF. Such bimodal information is fully exploited in the following hierarchical clustering in this study.

NMF in this study consists of the following steps:

1. The number of components *n*_*k*_ is assumed.
2. Matrix ***C*** is generated with its elements being non-negative random numbers.
3. $${\varvec{S}}=\left({\varvec{X}}{{\varvec{C}}}^{{\varvec{T}}}\right){\left({\varvec{C}}{{\varvec{C}}}^{{\varvec{T}}}\right)}^{-1}$$.
4. Negative values in ***S*** are set to zero. Each column vector of ***S*** is normalized.
5. $${\varvec{C}}={\left({{\varvec{S}}}^{{\varvec{T}}}{\varvec{S}}\right)}^{-1}\left({{\varvec{S}}}^{{\varvec{T}}}{\varvec{X}}\right)$$.
6. Negative values in ***C*** are set to zero. Each row vector of ***C*** is sorted by its *l*_2_ norm. The column vectors of ***S*** are also sorted according to the order of the corresponding row vectors of ***C***.
7. The mean square error (MSE) of the current estimation is calculated using ${\varvec{M}}{\varvec{S}}{\varvec{E}}={\left({n}_{xy}\times {n}_{uv}\right)}^{-1}\sum {\left({\varvec{X}}-{\varvec{S}}{\varvec{C}}\right)}^{2}$, and its convergence is judged by comparing it with the previous MSE. If the result does not converge, proceed to Step 3.
8. To survey the global minimum, i.e., to avoid local minima, the NMF is performed multiple times from Steps 2 to 7, and the minimum MSE with the corresponding matrices ***S*** and ***C*** are obtained.

This alternate least-square (ALS) NMF procedure^21^ is the same as that reported by us^19^, and specific DigitalMicrograph scripts for the NMF were presented as Supplementary Material (Sect. 1).

NMF has two technical difficulties: first is the possibility of convergence to a local minimum. To avoid local minima in this study, many computations were performed with different initial values (see Step 8 of the NMF procedure above) to find the optimal solution that yields the minimum MSE. The second issue is that the number of components, which is unknown, has to be assumed in advance for NMF (see Step 1). Although there is no established method to determine the number of components, we estimated the sufficient number of components *n*_*k*_ by comparing the MSEs of PCA and NMF, as shown in the following section. We assumed a sufficiently large value (*n*_*k*_ = 20), and the subsequent clustering allowed the validity of the selected value to be contemplated.

The normalization in Step 4 and the sorting in Step 6 are not necessarily required in general NMF algorithms, but they can improve the interpretability of the factorized diffractions and maps. In the actual experiment, the number of illuminated electrons is constant at each probe position, and the specimen thickness is nearly constant. Therefore, it is reasonable to normalize the scattering intensity of each diffraction in Step 4. The sorting of ***C*** and ***S*** in Step 6 helps to infer major components, as discussed below. The NMF was performed without regularization (which was implemented in the in-house script) because it does not lead to a substantial improvement.

### Outline of hierarchical clustering using crystallographic similarity

Clustering is a standard procedure in unsupervised machine learning, and several algorithms are available. Because the number of components is unknown, we used hierarchical clustering. The similarity between vectors in general clustering procedures is evaluated using Euclidean distances or cosine similarity, but such conventional measures are not related to the diffraction physics governed by Bragg’s law, $2d{\text{sin}}\theta =\lambda$, where *d* is the lattice distance, $\theta$ is the scattering angle, and $\lambda$ is the wavelength of electrons. The scattering angle $\theta$ is critical in crystallography because it is directly proportional to the inverse lattice constant of the material. Accordingly, we used another similarity based on diffraction physics to generate a nested set of clusters and to represent the hierarchical tree as a dendrogram.

A practical complication in actual experimental data is that the obtained diffraction patterns may be rotated by the rotation of the crystal domain in the specimen, even for the same crystal structure. We transformed diffractions ${\varvec{s}}(u,v)$ into $r-\phi$ projected patterns ${\varvec{s}}\boldsymbol{^{\prime}}\left(r,\phi \right)$, where *r* represents the scattering angle $\theta$ on the diffraction pattern and $\phi$ is the rotation angle. The similarity between the $r-\phi$ projected patterns was computed using cross-correlation, where a shift only along the $\phi$ axis was allowed. We evaluated the similarity between two patterns ${\varvec{s}}\boldsymbol{^{\prime}}\left(r,\phi \right)$ using such a cross-correlation, and a rotation in the plane $(u,v)$ of the diffractions ${\varvec{s}}(u,v)$ could be corrected. The cross-correlation varies from − 1 to 1, with 1 at the peak representing the perfect similarity, and the peak off-centering reflecting the misalignment. The $r-\phi$ projection is often used in diffraction analyses, and the cross-correlation is also used to calculate the similarity and relative shift between 2D patterns^22^. The comparison of Euclidean distances and a cross-correlation is discussed in the following section using experimental results.

### Experimental results and data preprocessing

**Figure 3:**
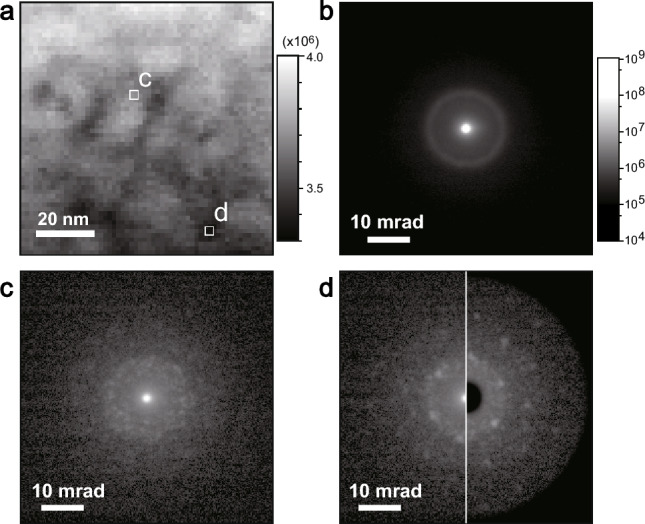
4D-STEM experimental results. (**a**) Virtual bright-field STEM image. (**b**) Integrated diffraction pattern of the entire scanning area. (**c**) and (**d**) Selected diffraction patterns obtained at positions (**c**) and (**d**) indicated in panel (**a**).

Figure 3a shows a bright-field image constructed from the 4D-STEM data using a virtual bright-field detector of 1 mrad in a semi-angle (= 2 nm^−1^) at the center of the diffractions, with ${{\varvec{c}}}_{2{\varvec{D}}}\left(x,y\right)=\sum_{\left|{u}^{2}+{v}^{2}\right|<1}{{\varvec{I}}}_{4{\varvec{D}}}$. We calculated an integrated diffraction pattern (Fig. 3b) from the entire area, ${{\varvec{s}}}_{2{\varvec{D}}}\left(u,v\right)=\sum_{x,y}{{\varvec{I}}}_{4{\varvec{D}}}$, in which the brightness of the diffraction is proportional to a logarithmic value of the signal intensity. The intensity of the center spot (i.e., the direct spot) shown in Fig. 3b was more than 100 times higher than that of the surrounding halo rings. Because of these halo rings, the specimen was considered to be amorphous. Since the scattering intensity of the halo rings is weak, less than 1/100 of the transmitted wave, the specimen thickness is considered to be sufficiently thin. The effect of dynamical diffraction can be approximately neglected, and the linear combination approximation of Eq. (1) is applicable. It should be noted that the nonlinear feature becomes evident in convergent beam electron diffraction in high-resolution STEM with a large convergence angle.

The diffraction patterns of the 4D-STEM data depend on the probe position, and Fig. 3c and d show selected diffractions obtained at positions c and d in Fig. 3a, respectively. Some areas present the regular spots of crystalline domains, as shown in Fig. 3d. Because the incident probe was illuminated coherently in small areas, the diffraction patterns exhibited speckle features that changed randomly with varying probe positions and were evident in the amorphous diffractions, as shown in Fig. 3c. A statistical approach is thus indispensable to distinguish regular crystalline spots from random speckle features.

The experimental data include subtle noise from the detection system and comparable quantum noise due to the limited number of electrons captured per pixel. Typically, hundreds of electrons are involved in each pixel, and it can contain tens of percent quantum noise according to the Poisson distribution. Although no normalization or denoising was applied, minimal data preprocessing was performed prior to NMF. Each diffraction pattern was accompanied by an intense direct spot at the center, and this high-intensity area became dominant for calculating the MSE; however, the direct spot is insensitive to the crystal structure. We used a mask to cover the intense direct spot (right half of Fig. 3d) to select the structure-sensitive area. Additionally, the four corners were roundly masked in advance for the $r-\phi$ projection.

### Dimensionality reduction of experimental results

**Figure 4:**
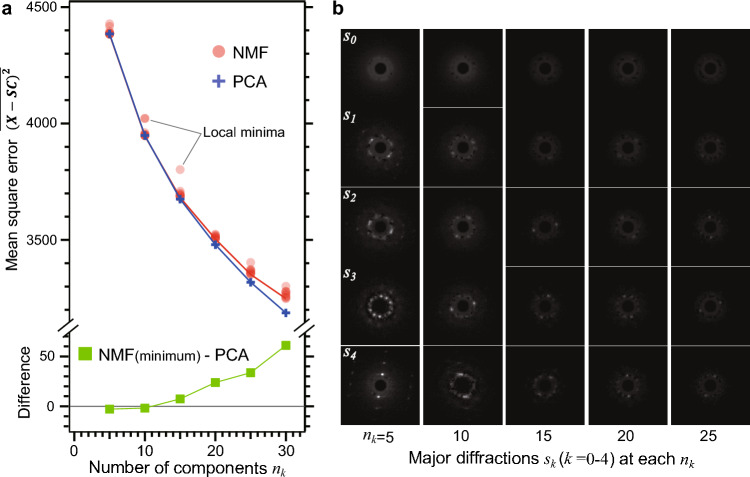
NMF results of the experimental 4D-STEM data with varying assumed number of components. (**a**) MSEs of NMF and PCA as a function of the number of components. (**b**) The five major components obtained at each assumed number of components *n*_*k*_.

We performed the NMF procedure on the experimental 4D-STEM data; the assumed number of components *n*_*k*_ was varied from 5 to 30. The MSEs of all converged NMFs are plotted as filled circles in Fig. 4a. Some points were considered to be converging to local minima; however, the monotonic decrease suggests that the minimum value of each assumed *n*_*k*_ was the optimal factorization. The MSEs of NMF and PCA deviated monotonically, and PCA exhibited a smaller MSE at a higher *n*_*k*_ (see filled squares in Fig. 4a). This deviation has been reported by our group and another group^4,19^. The deviation suggests that NMF cannot reproduce the experimental noise owing to the lack of a negative value. Figure 4b shows the NMF results at each* n*_*k*_, showing the five major diffraction patterns. We also found that as the number of components *n*_*k*_ increased, the multiple components can be considered to an amorphous matrix. We confirmed a similar *n*_*k*_ dependence using simulated data consisting of a known number of components, as shown in Sect. 5 of the Supplementary Material. Similar to Fig. 4a, the MSEs of the NMF for the simulated data became larger than those of the PCA when the assumed number became larger than the known number of components. We also found multiple amorphous components of the simulated data at higher *n*_*k*_ (see Fig. S5), as shown in Fig. 4b. Consequently, we considered *n*_*k*_ = 20 as a sufficient number of components to describe the experimental results.

**Figure 5:**
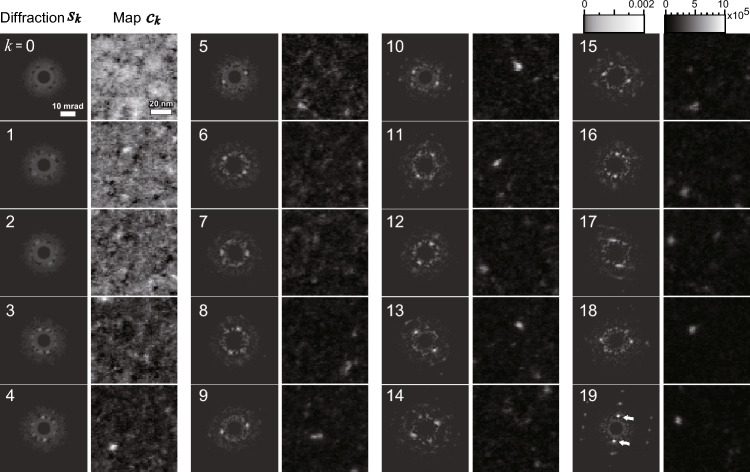
Diffraction *s*_*k*_ and map *c*_*k*_ pairs obtained using NMF (*n*_*k*_ = 20). The low intensity of diffractions is enhanced to visualize weak features. Diffractions in the linear intensity scale are shown in Fig. S9.

Figure 5 shows the NMF results obtained assuming *n*_*k*_ = 20. The matrices ***C*** and ***S*** providing the minimum MSE were fully transformed into diffraction ${{\varvec{s}}}_{{\varvec{k}}}\left(u,v\right)$ and map ${{\varvec{c}}}_{{\varvec{k}}}\left(x,y\right)$ pairs. The pairs were sorted according to the *l*_*2*_ norm of each map, as indexed from 0 to 19. The brightness of the diffractions and maps in Fig. 5 was set in the same lookup tables, in which low intensity of diffractions is enhanced to visualize the weak intensity features (see the intensity bar in the upper right of Fig. 5). Low-index (e.g., *k* = 0–3) components exhibited dispersed intensity in maps*** c***_*k*_ and no intense peaks in diffractions*** s***_*k*_, suggesting an amorphous matrix. By contrast, high-index (e.g., *k* = 9–19) components exhibited precipitates in the maps and crystalline spots in the diffraction patterns. The sorting roughly distinguished between an amorphous matrix and small crystalline precipitates.

Although NMF was performed without 2D information, factorized diffractions ${{\varvec{s}}}_{{\varvec{k}}}(u,v)$ exhibited symmetric spots as well as actual diffractions of thin specimens (e.g., white arrows on ***s***_**19**_). It should be noted that diffraction patterns of a thin crystalline specimen generally show symmetric spots due to the elongation of reciprocal lattice rods. The high-index maps exhibited a small precipitate with a diameter of several nanometers (e.g., 4 × 4 pixels). The factorized diffractions with symmetric spots are consistent with small crystalline precipitates observed in the maps. Because the adjacent pixels of the maps were independently processed in NMF, the precipitates detected in the maps were not artifacts due to speckle features but actual crystalline domains.

Some dark spots are seen in low-index diffractions (e.g., ***s***_***0***_–***s***_***2***_), and it is due to the assignment of some amorphous signals to crystalline components. This is a known artifact of NMF, the so-called unnatural drop of Shiga et al.^12^. The dark spots seen in Fig. 5 are faint, and they cannot be recognized in a linear intensity display, as shown in Fig. S9. Since the primary purpose of NMF is to extract the crystalline signals, these unnatural drops can be neglected.

As shown in Fig. 5, sorting by the *l*_*2*_ norm of the maps suggests that low-index components correspond to amorphous and high-index components to crystalline. However, it is difficult to quantitatively assess the degree of crystallinity from the intensity of crystalline spots (see Sect. 7 of the Supplementary Material). To interpret these NMF results crystallographically, the clustering described below is necessary.

### Similarities in factorized diffractions and maps

After dimensionality reduction by NMF, hierarchical clustering was performed for comprehensive nanostructural analyses. Evaluation of the similarity (or distance) between the components was required for clustering. We measured various similarity values in the diffractions ${{\varvec{s}}}_{{\varvec{k}}}\left(u,v\right)$ of matrix ***S*** and the maps ${{\varvec{c}}}_{{\varvec{k}}}\left(x,y\right)$ of matrix ***C*** to select a suitable function for hierarchical clustering in 4D-STEM.

**Figure 6:**
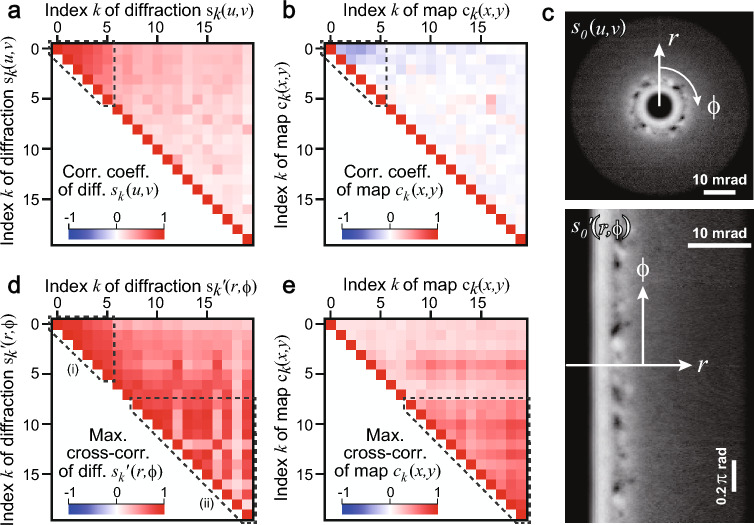
Similarity analyses using the correlation coefficient (1D) and cross-correlation (2D). (**a**) Correlation coefficients of diffractions ${{\varvec{s}}}_{{\varvec{k}}}\left(u,v\right)$. (**b**) Correlation coefficients of maps ${{\varvec{c}}}_{{\varvec{k}}}\left(x,y\right)$. (**c**) Example of *r*–*ϕ* projection of diffraction from ${{\varvec{s}}}_{0}\left(u,v\right)$ to ${{\varvec{s}}{\prime}}_{0}\left(r,\phi \right)$. (**d**) Maximum cross-correlation of diffractions ${{\varvec{s}}{\prime}}_{{\varvec{k}}}\left(r,\phi \right)$ disregarding shifts along the *r* axis. (**e**) Maximum cross-correlation of maps ${{\varvec{c}}}_{{\varvec{k}}}\left(x,y\right)$.

First, we calculated a well-known statistical parameter, the correlation coefficient, between 1D vectors. Figure 6a and b show the correlation coefficients in the 20 factorized diffractions ${{\varvec{s}}}_{{\varvec{k}}}\left(u,v\right)$ and maps ${{\varvec{c}}}_{{\varvec{k}}}\left(x,y\right)$, respectively. Figure S1a and b show tableaus of the scattering plots of the maps and diffractions. The low-index diffractions (dashed triangle in Fig. 6a) exhibited positive correlation coefficients (e.g., 0.84 between ${{\varvec{s}}}_{0}$ and ${{\varvec{s}}}_{1}$)), suggesting their similarities. The corresponding low-index maps (dashed triangle in Fig. 6b) exhibited weak negative correlation coefficients (e.g., − 0.26 between ${{\varvec{c}}}_{0}$ and ${{\varvec{c}}}_{1}$), suggesting a relative complementarity between the amorphous areas. The high-index (*k* > 9) diffractions and maps exhibited low correlation coefficients (Fig. 6a and b). Therefore, the correlation coefficient was unsuitable for clustering because it could not assess the similarity between the high-index crystalline diffractions. We also calculated other well-known distances, including the Euclidean distances and cosine similarity (Fig. S2); however, they were unsuitable for finding similarities between the high-index crystalline diffractions as well as the correlation coefficient. Consequently, the conventional statistical distances of diffractions can only distinguish amorphous and crystalline domains, and cannot reflect similarities in the crystalline diffraction patterns, which are indispensable for the clustering in this study.

Subsequently, we transformed the diffractions ${{\varvec{s}}}_{{\varvec{k}}}(u,v)$ into $r-\phi$ projected diffractions ${{\varvec{s}}\boldsymbol{^{\prime}}}_{{\varvec{k}}}\left(r,\phi \right)$. Figure 6c illustrates the projection for the first component (*k* = 0) from ${{\varvec{s}}}_{0}\left(u,v\right)$ to ${{\varvec{s}}\boldsymbol{^{\prime}}}_{0}\left(r,\phi \right)$ as an example. The *ϕ* rotation of diffraction ${{\varvec{s}}}_{{\varvec{k}}}(u,v)$ becomes a shift along the *ϕ* axis of ${{\varvec{s}}\boldsymbol{^{\prime}}}_{0}\left(r,\phi \right)$. We evaluated the maximum cross-correlations between ${{\varvec{s}}\boldsymbol{^{\prime}}}_{{\varvec{k}}}\left(r,\phi \right)$ while neglecting the lateral shift along the *r* axis (Fig. 6d). Contrary to the correlation coefficient of ${{\varvec{s}}}_{{\varvec{k}}}\left(u,v\right)$ (Fig. 6a), the maximum cross-correlations (Fig. 6d) exhibited variations in the high-index diffractions of ${{\varvec{s}}\boldsymbol{^{\prime}}}_{{\varvec{k}}}\left(r,\phi \right)$ (large triangle (ii) in Fig. 6d), that is, several high-index diffractions were similar after rotating the diffraction in the (*u*, *v*) plane. We performed clustering using this similarity measure in the following section.

To investigate the similarity of the real-space distribution, we can also calculate the maximum cross-correlations between maps ${{\varvec{c}}}_{{\varvec{k}}}\left(x,y\right)$ (Fig. 6e) considering 2D lateral (*x*, *y*) shifts. Unlike the conventional correlation coefficient (Fig. 6b), the cross-correlation exhibited high similarities in the high-index maps (triangle in Fig. 6e), which suggests that the crystalline precipitates have a similar shape but are located at different positions (see the maps ${{\varvec{c}}}_{{\varvec{k}}}(x,y)$ in Fig. 5). The differences between Figs. 6a and 6d and that between Figs. 6b and e were due to the difference between the correlation coefficient and cross-correlation. Consequently, the 2D cross-correlation, as compared to the correlation coefficient of the 1D vectors, provided additional information.

### ***Hierarchical clustering using cross-correlation of ***${\varvec{r}}-{\varvec{\phi}}$*** projected diffractions***

The factorized results were hierarchically clustered based on the crystallographic similarities of diffractions, as mentioned above. We wrote DigitalMicrograph scripts for the clustering from scratch, and all the Python libraries (e.g., Matplotlib^23,24^) were only used for drawing dendrograms. The written clustering steps were as follows: (a) select the two most similar diffractions from all diffraction components using cross-correlations of the ${\varvec{r}}-{\varvec{\phi}}$ projected diffractions; (b) calculate an integrated diffraction from the two selected diffractions after correcting for each $\phi$ rotation; (c) reconstruct all diffraction components from the diffractions not selected in Step a and the integrated diffraction in Step b; and (d) return to Step a until all diffractions are clustered.

**Figure 7:**
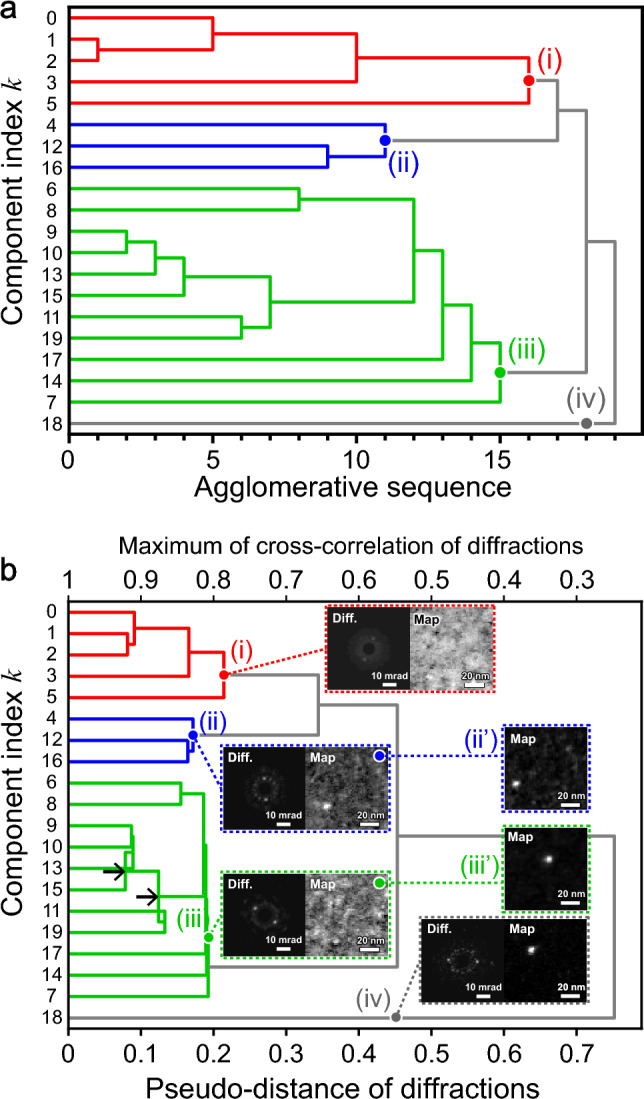
Dendrograms constructed based on the similarity of diffractions. (**a**) Dendrogram as a function of clustering sequence. (**b**) Dendrogram as a function of pseudo-distance (1- cross-correlation). Insets indicate the average diffractions and maps of groups i–iv. Diffraction rotations were corrected. Maps ii and iii are simple summations without shift correction, whereas maps ii’ and iii’ are integrated results with shift correction to visualize average precipitate shapes.

Figure 7 shows dendrograms drawn using Matplotlib in DigitalMicrograph. Dendrograms are generally drawn along the component index and distance axes. To clarify clustering, we first illustrate a dendrogram in which the horizontal axis is not a distance but an agglomerative sequence from 0 to 19 (Fig. 7a). Another dendrogram (Fig. 7b) with the pseudo-distance along the horizontal axis was also generated in accordance with conventional dendrograms. Since the cross-correlation between two diffractions becomes the maximum of one for the exact match diffractions, we defined the pseudo-distance as the deviation from one (i.e., 1 − cross-correlation value). The pseudo-distances did not monotonically increase in the agglomerative sequence (see two arrows in Fig. 7b) possibly owing to the reduction in quantum noise in the integrated diffraction. In other words, we can improve the signal-to-noise ratio using clustering, which is convenient for actual experimental data in which quantum noise is inevitable.

Cross-correlation is commonly used to determine the similarity between 2D patterns. Based on the cross-correlation values, we identified four groups (i.e., clusters) i–iv, as shown in Fig. 7b. Groups i–iv consisted of components (0, 1, 2, 3, 5), (4, 12, 16), (6, 8, 9, 10, 13, 15, 11, 19, 17, 14, 7), and (18), respectively.

Insets i–iv in Fig. 7b show the integrated diffractions and maps of the four groups. Group i represents amorphous areas, whereas the remaining groups represent crystalline precipitates. The three crystalline groups, ii–iv, were distinguished by the Bragg angle of the diffraction spots. The diffraction spots of groups ii–iv corresponded to lattice distances of 0.259, 0.228, and 0.191 nm, respectively. This suggests that the three diffraction spots should be examined as the first phase of crystalline precipitation. It should be noted that no spot was recognized in the conventional diffraction (Fig. 3b) integrated from the entire area. Identifying the three crystalline precipitate groups from the 3364 experimental diffractions would be challenging without the optimized unsupervised machine learning.

The integrated maps i–iv shown in Fig. 7b are summations without relative-shift correction; therefore, the precipitates were randomly distributed and their shapes were unclear. Because relative shifts could be measured using cross-correlation (see Fig. 6e), we calculated the shift-corrected integrated maps of groups ii and iii, as shown in maps ii’ and iii’ of Fig. 7b. We found that the typical grain size of the precipitates was approximately 7 nm in diameter, which is difficult to quantify using conventional STEM (see Sect. 6 of the Supplementary Material).

4D-STEM provides bimodal data, such as real-space maps and reciprocal-space diffractions. Although clustering (Fig. 7) was performed using the diffraction similarities, the map similarities could also be used for clustering, as shown in Fig. S3, where no group appeared within a short pseudo-distance. Therefore, diffractions, as compared to maps, seem to be advantageous for crystallographic analysis.

## Discussion

### Material characterization based on clustered groups

We analyzed the nanostructure of high-pressure annealed metallic glass Zr-Cu-Al^25^. Metallic glasses have attracted substantial interest because of their properties such as high strength and significant elastic elongation. As the inhomogeneity of a crystal structure is related to its material properties, structural changes using various treatments have been studied. However, nanometer-sized precipitates are difficult to detect using conventional diffractometry (e.g., electron or X-ray diffraction). The early stage of crystalline precipitation in various amorphous materials is an essential topic in crystallographic studies and for evaluating various material properties such as mechanical strength.

We elucidated the observed crystalline peaks in Fig. 7 based on several known phases, including ZrCu (B2), AlCu_2_Zr, Zr_2_Cu, and ZrCu (B19’, ICSD 167,596). The B19’ structure yields the following peaks: 0.254, 0.227, and 0.193 nm of *d*_110_, *d*_102_, and *d*_021_, corresponding to the 0.259, 0.228, and 0.191 nm peaks of groups ii, iii, and iv, respectively. All the crystalline precipitates were identified as the B19’ phase. The B19’ phase has been observed at the sub-micrometer scale^26^; therefore, our results indicate the early stage of crystalline precipitates. These nanometer-order precipitates are thought to be the seeds for further grain growth. It should be emphasized that NMF and 4D-STEM could statistically analyze all diffraction patterns. Therefore, our proposed structural analysis is comprehensive and quantitative while avoiding bias due to a small sampling.

### Detection limit and validation of NMF using simulated data

The minimum size of the crystalline precipitates detectable by 4D-STEM is comparable to the incident probe size. The precipitates inclined from the low-index axes could not be detected owing to the lack of clear diffraction spots; however, 4D-STEM, as compared to other electron microscopy techniques, effectively detects crystalline precipitates. Various other microscopic techniques (e.g., STEM/TEM imaging) are available to assess nanostructures (e.g., crystallinity). In the case of high-resolution STEM/TEM imaging, crystalline precipitates may be missed if they are out of focus due to the short depth of focus ($\lambda /{\alpha }^{2}$). Low-resolution STEM/TEM techniques may not be able to resolve the planar spacing along the high-index axes. By contrast, 4D-STEM can easily detect higher-order crystalline reflections and amorphous halo rings simultaneously. For small particles, the diffraction rods in the reciprocal space are elongated, reducing the likelihood of missing electron diffraction spots. In the present study, the presence and diameter of crystalline precipitates are unknown a priori; therefore, a robust and statistical analyses such as 4D-STEM should be used first.

Although NMF is an established technique for dimensionality reduction, we have validated our NMF procedure using simulated data with quantum noise, as described in Sect. 5 of the Supplementary Material. Even in the presence of severe quantum noise, the crystalline domains with weak signals (1%) were detected by the NMF. When a sufficient number of components that are larger than the simulated components is assumed, the NMF estimates multiple amorphous domains (Fig. S5). Additionally, the MSE of NMF becomes higher than that of PCA at a sufficient number of components. These features are similar to the abovementioned NMF of the experimental results in Fig. 4, suggesting that the assumed number (*n*_k_ = 20) of components is sufficient for the experimental results. Further discussion on the detectability and linearity of the NMF procedure is provided in Sect. 5 of the Supplementary Material.

In summary, we combined 4D-STEM and unsupervised machine learning for nanostructural analysis. Dimensionality reduction of experimental diffractions (3364) was achieved using NMF, resulting in sparse (20) interpretable diffractions and maps. Hierarchical clustering was performed based on the similarity between electron diffractions using 2D cross-correlations of $r-\phi$ projected diffractions instead of conventional Euclidean distances for 1D vectors. We obtained bimodal information from clustering, namely rotation-corrected diffractions and shift-corrected maps. Based on the dendrogram, we identified four groups, i.e., one amorphous and three crystalline groups with different diffraction spots. The three rotation-corrected diffraction patterns with different diffraction spots allowed the crystal structure of the precipitates to be identified. We also performed a real-space analysis of the three crystalline groups and evaluated the average particle size using the shift-corrected map of each crystalline group. Such investigations could not be achieved without combining 4D-STEM and unsupervised machine learning optimized with the knowledge of microscopic and diffraction techniques. This combination enables comprehensive bimodal real and reciprocal analyses, and it is applicable to various materials, such as metals, ceramics, and polymers.

## Methods

We analyzed a metallic glass Zr_50_Cu_40_Al_10_ specimen subjected to high-pressure (5.5 GPa) and high-temperature (880 K) treatments. The detailed structural and mechanical properties of the specimen are provided in our previous report^27^. A specimen for 4D-STEM was prepared by mechanical polishing and Ar ion milling (PIPS-II, Gatan) at 2 kV or less. We performed a 4D-STEM experiment using an electron microscope (Titan, Thermo Fisher Scientific) at an accelerating voltage of 300 kV. The 4D-STEM data were obtained in an 87 × 87 nm^2^ area using a 1.5 nm scan step (58 × 58 pixels) and a diffraction of 128 × 128 pixels (i.e., ${I}_{4D}\in {\mathbb{R}}^{58\times 58\times 128\times 128}$). We realized a small convergence semi-angle of 0.5 mrad using a small 0.5 μm diameter aperture (i.e., high angular resolution), and we were able to clearly distinguish crystalline spots from amorphous halo rings. The spatial resolution of the present 4D-STEM experiment depended on the diffraction limit, and the probe had a 2 nm full width at half maximum. The scan step of 1.5 nm was optimized to the probe size. Diffractions were acquired with an exposure time of 10 ms using a charge-coupled device detector (US1000 series, Gatan), and its intensity was converted into the number of electrons. The probe deflection system of the electron microscope was carefully aligned, and the center spot position of diffractions (see Fig. 3b) was stabilized during incident probe scanning using de-scanning coils.

We processed all data using the DigitalMicrograph software (Gatan), which allows the electron microscope system to be controlled and data to be analyzed through various functions (e.g., slicing, matrix operations, cross-correlation) and programming statements (e.g., for loops)^22,28^. Although Python is implemented in the latest version of DigitalMicrograph (above GMS 3.4), we prepared in-house DigitalMicrograph scripts from scratch for preprocessing, slicing (e.g., 4D to 2D data transformation), NMF, sorting, hierarchical clustering, data conversion, and other data processes. The present NMF procedure followed the procedure reported^21^. Our DigitalMicrograph script, which is simple, as compared to modern NMF algorithms^29^, is presented in Sect. 1 of the Supplementary Material; however, obtaining fast computational algorithms was not within the scope of this study. Instead, we aimed to combine a modern scientific instrument with data science based on the knowledge of characterization techniques; our in-house scripts provide transparency and enable customization. We also used the SciPy^30,31^ and Matplotlib^23,24^ libraries to draw dendrograms using Python implemented in DigitalMicrograph.

### Supplementary Information

Supplementary Information.

## Data Availability

The datasets generated during this study are available from the corresponding author upon reasonable request.

## Code availability

The part of codes for unsupervised machine learning used to perform this study are described in Sect. 1 of the Supplementary Material.
