# Supplementary material for: Unsupervised machine learning combined with 4D scanning transmission electron microscopy for bimodal nanostructural analysis
Source: Sci Rep. 2024 Feb 5;14:2901. doi: 10.1038/s41598-024-53289-5 (PMC11303778; doi:10.1038/s41598-024-53289-5)
Supplement: Supplementary file 1 — Supplementary Information. [file 41598_2024_53289_MOESM1_ESM.docx]

Supplementary Material

Unsupervised machine learning combined with 4D scanning transmission electron microscopy for bimodal nanostructural analysis

Koji Kimoto^a*^, Jun Kikkawa^a^, Koji Harano^a^, Ovidiu Cretu^a^, Yuki Shibazaki^b^, and Fumihiko Uesugi^c^

^a^ Center for Basic Research on Materials, National Institute for Materials Science, Tsukuba, Japan

^b^ Institute of Materials Structure Science, High Energy Accelerator Research Organization, Tsukuba, Japan

^c^ Research Network and Facility Service Division, National Institute for Materials Science, Tsukuba, Japan

*Corresponding author: Koji Kimoto

[kimoto.koji@nims.go.jp](mailto:kimoto.koji@nims.go.jp)

Center for Basic Research on Materials, National Institute for Materials Science (NIMS), 1-1 Namiki, Tsukuba, Ibaraki 305-0044, Japan

Unsupervised machine learning combined with 4D scanning transmission electron microscopy for bimodal nanostructural analysis

This supplementary material contains the following sections:

1. Nonnegative matrix factorization (NMF) code for DigitalMicrograph;
2. Scattering plots of factorized maps and diffractions;
3. Various distances measured between factorized diffractions and maps;
4. Hierarchical clustering using similarity between maps;
5. Validation of NMF using simulated data;
6. Conventional analysis of 4D-STEM data; and
7. Detailed feature and crystallinity of factorized diffractions.

# NMF code for DigitalMicrograph

We prepared a few DigitalMicrograph scripts for this study. DigitalMicrgraph software can be downloaded from the manufacturer's website (Gatan, Inc.). The NMF algorithm consisting of eight steps in this study is described in the main text. The core steps for NMF can be written using DigitalMicrograph functions as follows:

C = UniformRandom()

CT = MatrixTranspose(C)

CCT = MatrixMultiply(C, CT)

ICCT = MatrixInverse(CCT)

XCT = MatrixMultiply(X, CT)

S = MatrixMultiply(XCT, ICCT)

S = tert(S<0, 0, S)

ST=MatrixTranspose(S)

STS = MatrixMultiply(ST,S)

ISTS = MatrixInverse(STS)

STX = MatrixMultiply(ST, X)

C = MatrixMultiply(ISTS, STX)

C = tert(C<0, 0, C)

MSE = MeanSquare(X - MatrixMutiply(S, C))

# Scattering plots of factorized maps and diffractions

We calculated the correlation coefficients of twenty factorized maps and diffractions to illustrate the conventional statistical analysis (see Figs. 6a and 6b of the main paper). Figures S1a and S1b show the scattering plot tableaux for the maps and diffractions, respectively. We prepared a DigitalMicrograph script to draw these tableaux.


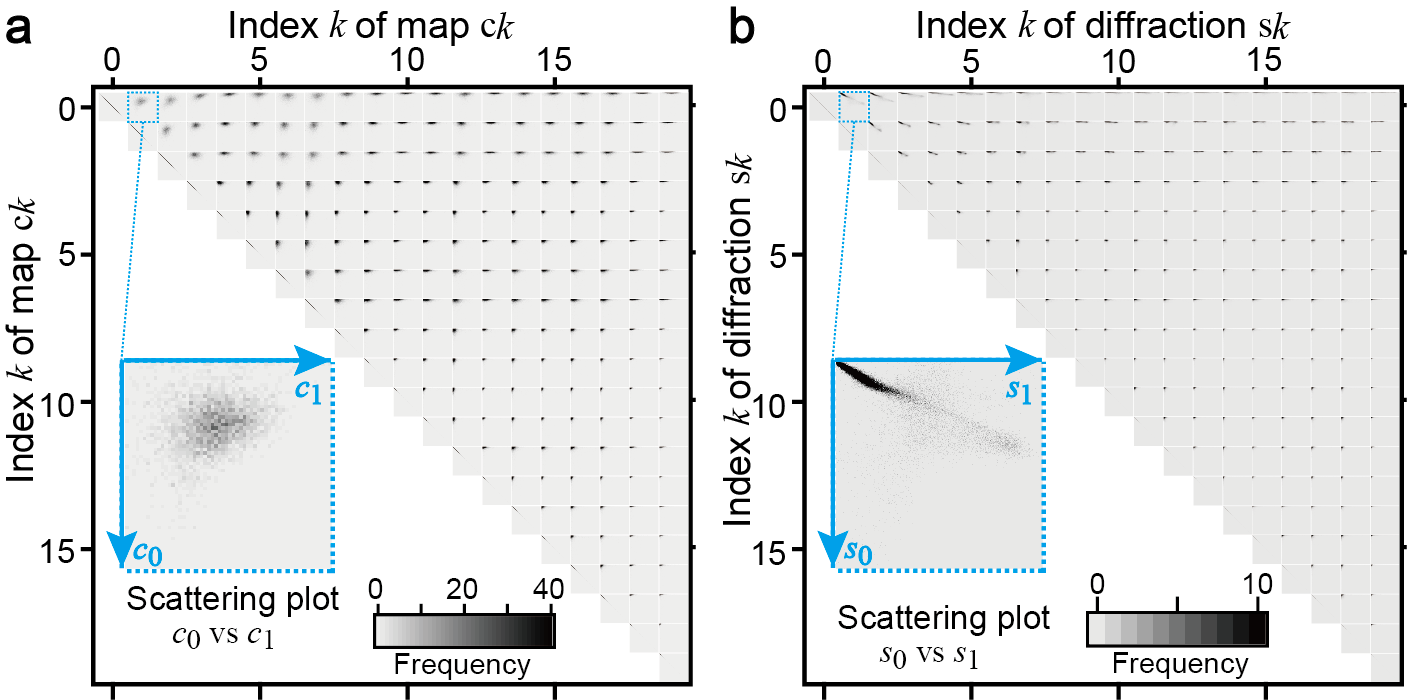


**Fig. S1 Tableaux of scattering plots of factorized maps and diffractions. a** Tableau of scattering plots of maps $\boldsymbol{c}_{\boldsymbol{k}}$. **b** Tableau of scattering plots of diffractions $\boldsymbol{s}_{\boldsymbol{k}}$. Insets show the enlarged scattering plots of the first (*k* = 0) and second (*k*= 1) components.

# Various distances measured between factorized diffractions and maps

The evaluation of similarities in the diffractions and maps is critical for hierarchical clustering. Several similarity measures are available for one-dimensional (1D) vectors, such as Euclidean distances and cosine similarities, and were calculated for maps, diffractions, and *r–φ* projected diffractions determined using NMF. Figure S2 shows three kinds of similarities, namely, Euclidean distance (left column), cosine similarity (middle column), and maximum cross-correlation (right column).

The Euclidean distance and cosine similarity can be used to divide low- and high-index maps and diffractions as well as the correlation coefficient (Figs. 6a and 6b). There was no notable difference between the measures for $\boldsymbol{s}_{\boldsymbol{k}}\left( u,v \right)$ and $\boldsymbol{s'}_{\boldsymbol{k}}\left( r,\phi\right)$ because both parameters were calculated between 1D vectors. Compared with the maximum cross-correlation (Fig. 6e), Fig. S2c shows additional negative peaks at low-index components because low-index maps show negative peaks owing to the complementary distribution of amorphous areas. We performed clustering using *r–φ* projected diffractions constrained only to the *φ*-axis shift (i.e., rotation on the (*u*, *v*) plane). Without such a constraint, almost all cross-correlations of diffractions become close to 1 (Fig. S2i) because diffraction spots at different Bragg angles look similar using *r*-axis shifting, and such a shift is meaningless in diffraction physics.


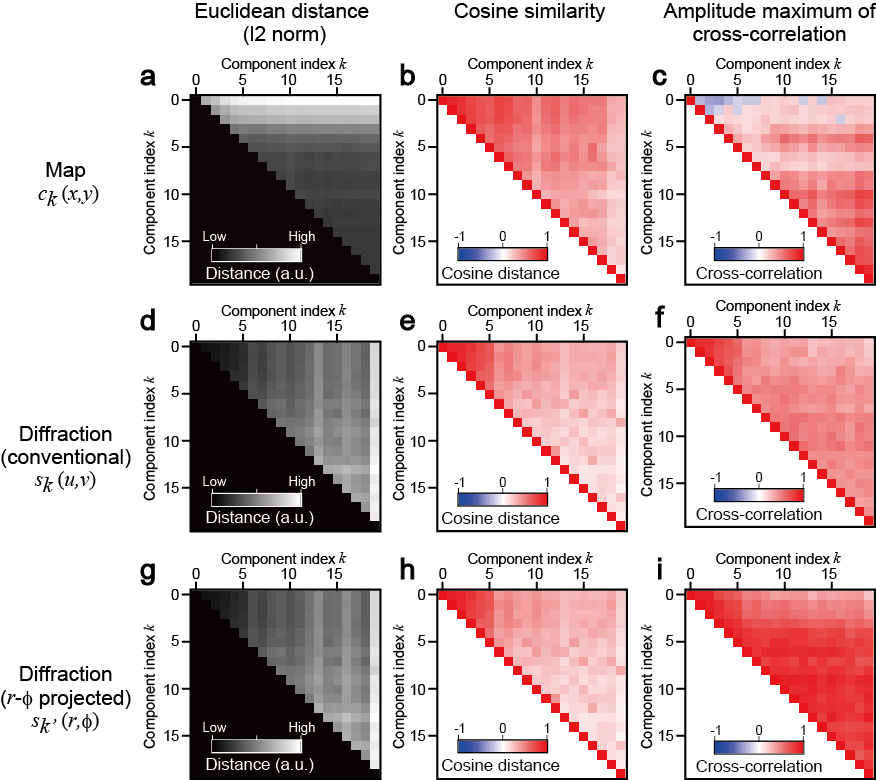


**Fig. S2 Various similarity analyses of maps, diffractions, and** $\boldsymbol{r-\phi}$ **projected diffractions** $\boldsymbol{s}_{\boldsymbol{k}}^{\boldsymbol{'}}$. Calculated Tableaux of **a,b,c** maps $\boldsymbol{c}_{\boldsymbol{k}}$, **d,e,f** diffractions $\boldsymbol{s}_{\boldsymbol{k}}$, and **g,h,i** $r-\phi$ projected diffractions $\boldsymbol{s}_{\boldsymbol{k}}^{\boldsymbol{'}}$. The figure shows Euclidean distances (left column), cosine similarities (middle column), and maximum cross-correlations (right column).

# Hierarchical clustering using similarity between maps

4D-STEM data provides real-space maps and reciprocal-space diffractions. In the main paper, we performed hierarchical clustering using similarities in diffractions, as shown in Fig. 7. Clustering can also be performed using similarities in maps, as shown in Fig. S3. The pseudo-distance (horizontal axis in Fig. S3) is the amplitude maximum of cross-correlations (Fig. 6e); therefore, the spatial drift was corrected. Because the amorphous areas exhibited negative cross-correlation peaks (Fig. S2c), clustering was performed using amplitude maxima.

The dendrogram (Fig. S3) based on map similarities exhibited differences from that based on diffraction similarities (Fig. 7b). Most clustering in the diffraction-based dendrogram occurs up to an approximate pseudo-distance of 0.2 (cross-correlation of 0.8). In contrast, the map-based dendrogram (Fig. S3) does not exhibit a large group within a short pseudo-distance. Therefore, the similarities between maps are unsuitable for clustering, even for precipitates. The diffraction-based approach is advantageous for crystallographic analysis.


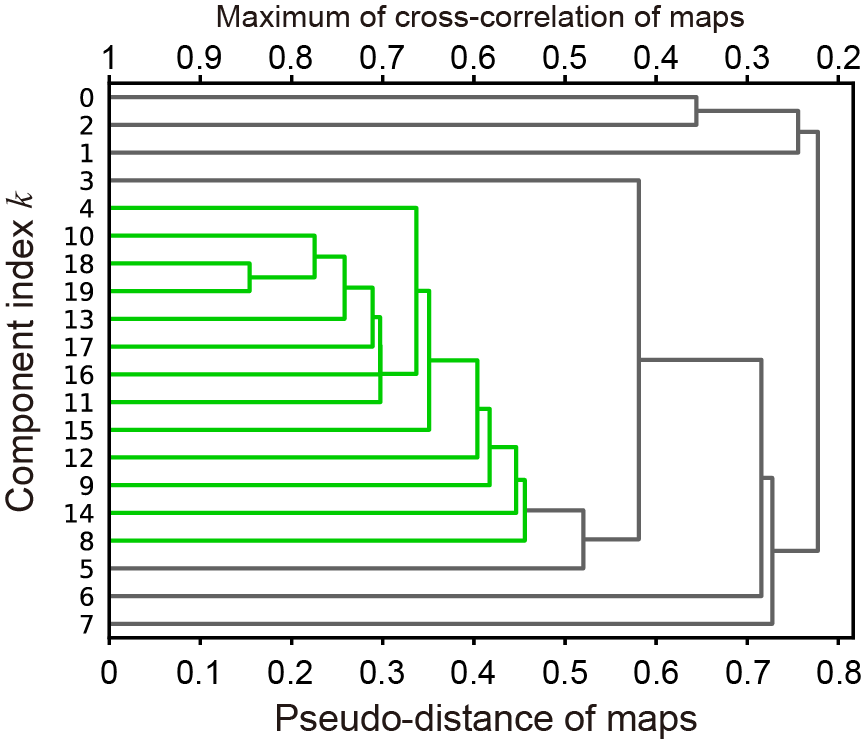


**Fig. S3** **Dendrograms constructed using the similarities of maps.** The drifts in the maps were corrected.

# Validation of NMF using simulated data

Although we applied the NMF to actual experimental data in the main paper, we validated the NMF on known simulated data with quantum noise. We constructed a simulated 4D-STEM data consisting of one amorphous and three different diffraction patterns (i.e., the number of components *n_k_* is four) with dimensions (*n_x_,n_y_,n_u_,n_v_*) = (36,36,128,128) was constructed. The real space (*x,y*) model contained nine crystalline domains (6 × 6 pixels each) in an amorphous matrix (Fig. S4a). The crystalline ratio *R_c_* of the nine domains varied between 50, 9, and 1%. Examples of each diffraction pattern are shown in Fig. S4b, where the left half represents ideal diffraction and the right half is the simulated data with quantum noise. The Poisson noise was implemented based on the number of electrons, *N* = 10^6^, in each diffraction. The condition of 10^6^ electrons per diffraction is of the same order as that obtained with a 2 pA probe current and 1 ms exposure time and is a practical experimental setting. As shown in Fig. S4b, diffraction spots were visible when the amorphous to crystalline ratio *Rc* was 50% (i, iv, vii) and 9% (ii, v, viii); however, diffraction spots were more difficult to detect when the ratio was 1% (iii, vi, ix) due to severe quantum noise.


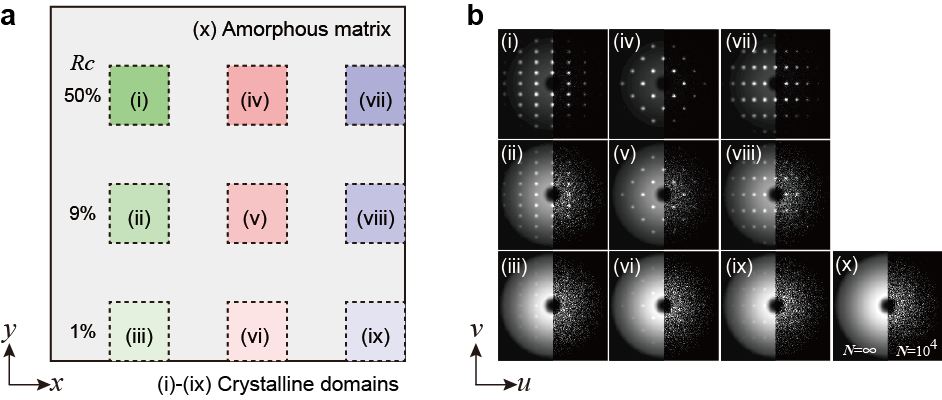


**Fig. S4** **Simulated 4D-STEM data to validate the NMF procedure.** **a** Real space structure of the simulated data. **b** Diffraction examples from areas (i)–(x).

We ran NMF ten times each, assuming the number of components to be *n_k_* = 4, 5, 6, 10, 20, and 30. Note that the actual number of components was four. The mean square errors (MSEs) of all converged NMF results are plotted as filled circles in Fig. S5a (similar to Fig. 4a). The MSEs of the NMF and principal component analysis (PCA) deviated monotonically, and NMF exhibited larger MSEs at higher *n_k_* (see filled square in Fig. S5a). This deviation suggests that the NMF cannot reproduce the experimental noise due to the lack of a negative value. Figure S5b shows the NMF results at each *n_k_* with the major (low index) five diffractions shown. A sufficient number of elements resulted in multiple amorphous matrix components.

It should be noted that these multiple amorphous components (Fig. S5b) are similar to the experimental results shown in Fig. 4b. Since the experimental results in Fig. 5 (*n_k_* = 20) exhibited multiple amorphous components, we consider that the assumed number (*n_k_* = 20) was sufficient to represent the experimental results.


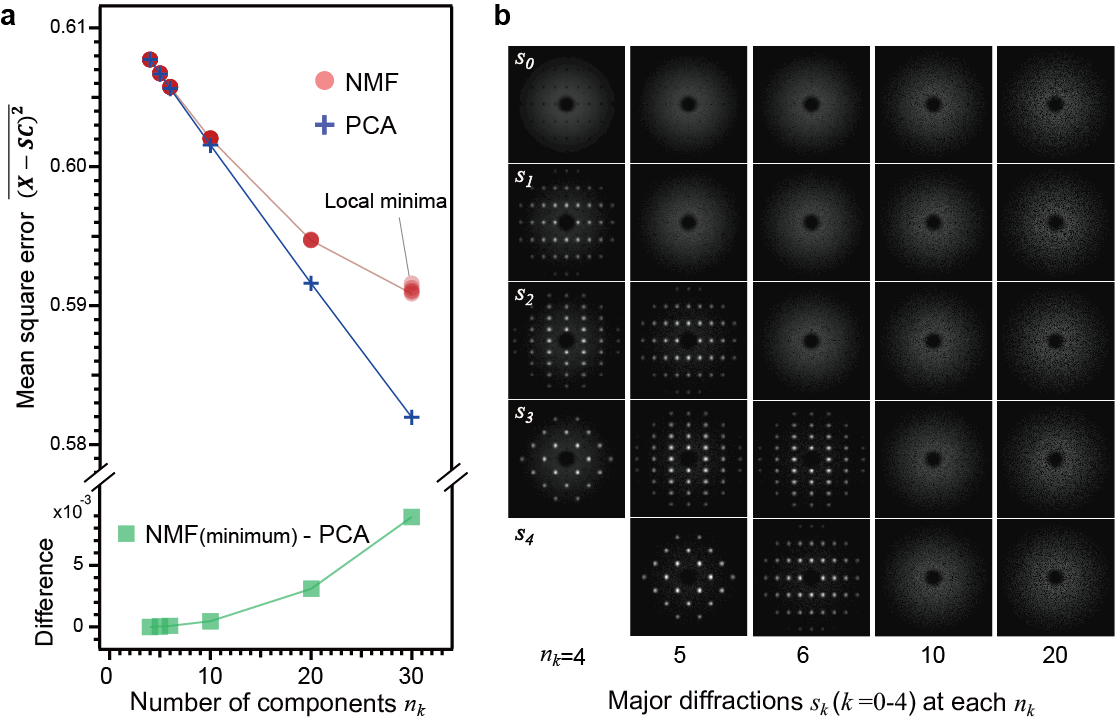


**Fig. S5 NMF results of simulated 4D-STEM data with varying assumed number of components. a** MSEs of NMF and PCA as a function of the number of components. **b** The major five components obtained at each assumed number of components *n_k_*.

Figures S6a and S6b respectively show the PCA and NMF results at *n_k_* = 6. PCA correctly estimated four principal components and two additional noises that included negative values. In contrast, the NMF results exhibited three amorphous and three crystalline components. The correlation coefficient (Fig. S6c) of the NMF results shows the similarity in *k* = 0–2, as also shown in the main paper and Fig. 6a. The amorphous matrix was divided into three components of diffractions and maps due to the assumed large number (6) beyond the actual number (4) of components.

Maps ***c*_3_**, ***c*_4_**, and ***c*_5_** of Fig. S6b show crystalline domains even in (iii), (vi), and (ix), where the crystal concentration was the lowest (1%) (see white arrows in Fig. S6b). The nominal and estimated crystal ratios and their pixel-by-pixel variation (standard deviation) are plotted as markers and error bars, respectively, in Fig. S7. Although NMF detected all crystalline components, the quantitative comparison exhibited systematic deviations, for example, 50% of the crystalline domain was estimated to be 60% (Fig. S7a). We speculate that it is due to estimation errors of the crystalline components in which amorphous backgrounds are included. The high detection sensitivity for weak crystalline signals is noteworthy, as shown in Fig. S7b. The estimated crystalline ratios were higher than those of the amorphous matrix and its standard deviation, indicating the superior detectability of the NMF procedure.


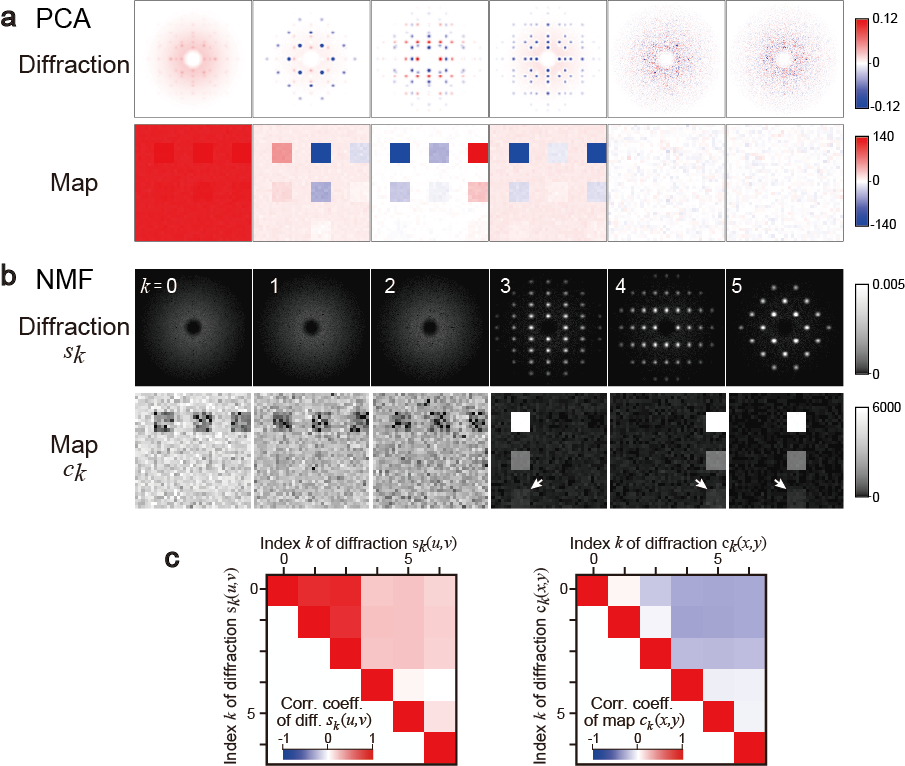


**Fig. S6 PCA and NMF results of the simulated 4D-STEM data.** **a** Principal components using PCA. **b** NMF results assuming *n_k_* = 6. **c** Calculated Tableaux of the correlation coefficients of diffractions and maps obtained using the NMF analysis.


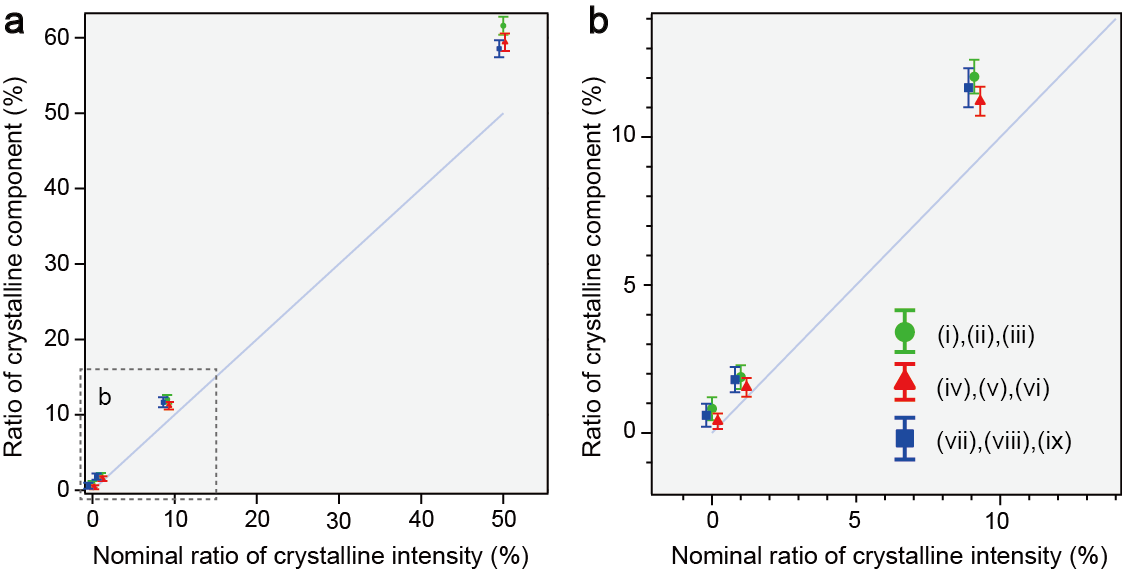


**Fig. S7** **Quantitative comparison between the nominal and estimated crystalline ratios on nine crystalline domains (i)–(ix) and the amorphous matrix (x).**

In summary, we investigated the validity of our NMF using simulated 4D-STEM data with significant quantum noise, demonstrating that NMF was sufficiently robust to detect weak crystalline components. The MSEs of PCA and NMF were quantitatively clarified, and the sufficient number of components could be estimated by calculating the difference between the MSEs of PCA and NMF.

# Conventional analysis of 4D-STEM data

Crystallographic information can also be obtained from 4D-STEM data without machine learning techniques. To clarify the progress of the combination with the optimized machine learning techniques, we demonstrate the limitations of conventional methods (e.g., virtual annular dark-field (ADF) imaging) applied to the experimental 4D-STEM data.

We constructed virtual ADF images using a very narrow scattering angle range to distinguish between crystalline and amorphous regions. Figures S8a–8d present virtual ADF images with varying angle ranges. The corresponding virtual bright-field image and integrated diffraction are shown in Figs. S8e and 8f, respectively. The contrast in the virtual ADF images was reversed depending on the scattering angle, indicating material inhomogeneity. However, it was challenging to determine which parts corresponded to crystalline precipitates. Crystalline particles could be distinguished, but the averaged shape and diameter of the crystalline precipitates could not be evaluated. In contrast, the present combination of 4D-STEM and machine learning allows individual particles to be isolated and provides an average representation of their crystallographic features.


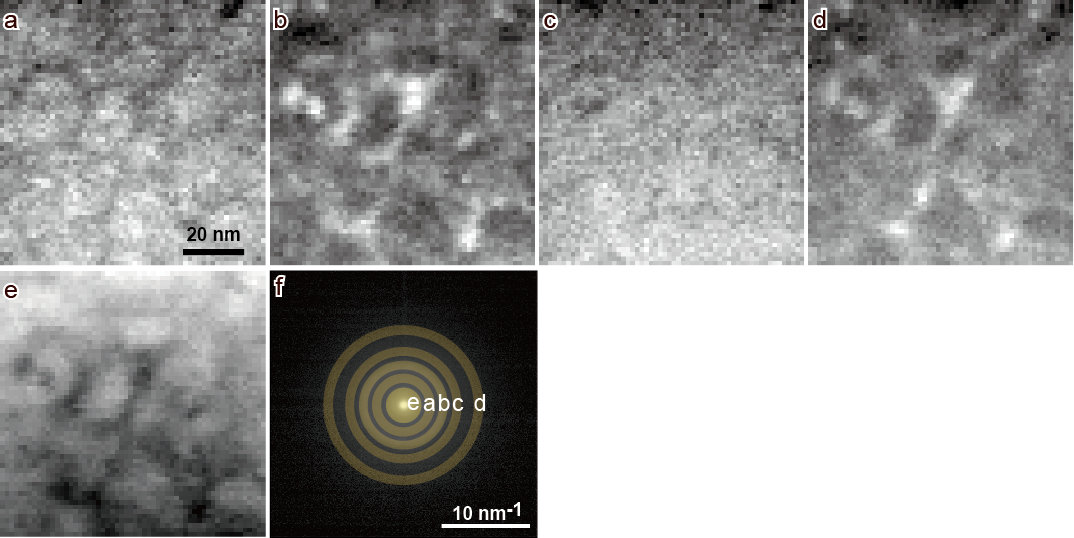


**Fig. S8. Conventional analysis of 4D-STEM data.** Virtual annular dark-field images constructed using different scattering angles: **a** 2.5–3.5, **b** 4.0–5.0, **c** 5.5–6.5, and **d** 8.0–9.0 nm^-1^. **e** Corresponding virtual bright-field image (0–2 nm^-1^). **f** Diffraction pattern and corresponding scattering angle ranges for the virtual imaging.

# Detailed feature and crystallinity of factorized diffractions

As shown in Fig. S5, it is possible to distinguish crystal diffractions from amorphous ones in simulated data, but it is not easy in experiments (Figs. 4 and 5). We consider that the best way to classify diffractions as crystal or amorphous is clustering, as demonstrated in our study.

In Figs. 4 and 5, the logarithm of intensity is shown as brightness to visualize low-intensity features in diffraction. Figure S9a shows the same diffractions from Fig. 5 in the linear intensity scale. It is clear that only high-index diffractions show crystalline spots.

To our knowledge, there is no established criterion for the quantitative determination of crystallinity based on diffraction patterns. Since amorphous materials are assumed to have azimuthally-constant intensities, we quantified the crystalline intensity ratio of the NMF results, as shown in Fig. S9b and S9c. Although the high-index diffractions show high maxima and high crystalline intensity ratios, these do not provide us with a discriminating criterion.


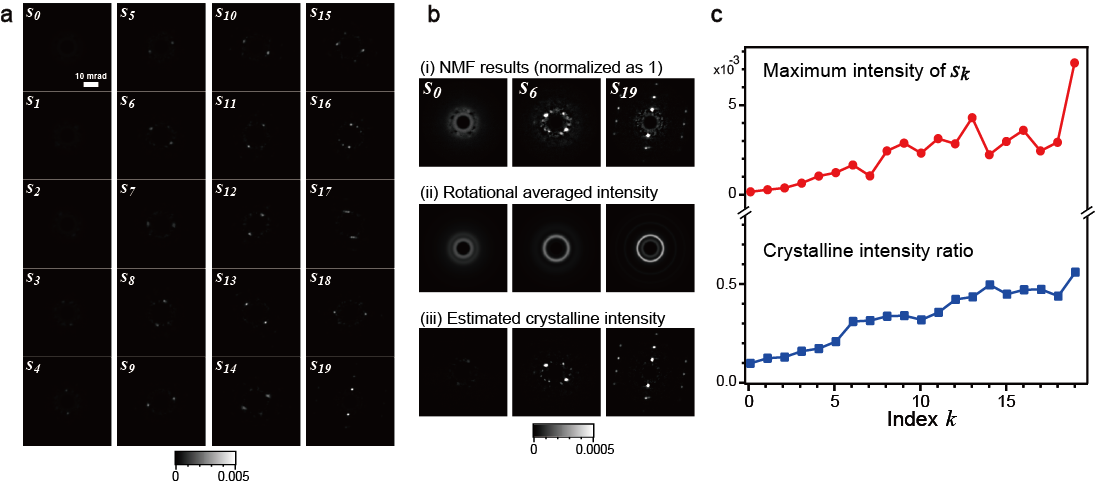


**Fig. S9 a** Diffractions ***s_k_*** in linear intensity scale obtained using NMF (see also Fig. 5). Note that each diffraction was normalized so that the integrated intensity was 1.

**b** Procedure for estimating crystalline intensities. The rotationally-averaged intensity (ii) was subtracted from each NMF result (i), and positive values were considered as crystalline intensity (iii). **c** Index *k* dependencies of maximum intensity and crystalline intensity ratio.
